# Studying of anti‐inflammatory and antioxidant effects of tectorigenin in ovalbumin‐induced asthma mice models

**DOI:** 10.1111/crj.13742

**Published:** 2024-04-25

**Authors:** Jingning Guo, Yanping Shi, Yujun Wang, Seyyed Shamsadin Athari, Tao Chen

**Affiliations:** ^1^ Department of Integrated Traditional Medicine and Western Medicine Xi'an Children's Hospital Xi'an China; ^2^ Department of Immunology, School of Medicine, Zanjan University of Medical Sciences Zanjan Iran

**Keywords:** allergy, Chinese medicine, herbal medicine, inflammation

## Abstract

**Background:**

Allergic asthma is an important respiratory system problem characterized by airway inflammation, breathlessness, and bronchoconstriction. Allergic asthma and its outcomes are triggered by type 2 allergic immune responses. Tectorigenin is a methoxy‐isoflavone with anti‐inflammatory effects. In this study, we investigated the effects of tectorigenin on the pathophysiology of allergic asthma in an animal model.

**Methods:**

Asthmatic mice were treated with tectorigenin. Then airway hyperresponsiveness (AHR), eosinophil percentage, levels of interleukin (IL)‐33, IL‐25, IL‐13, IL‐5, IL‐4, total and ovalbumin (OVA)‐specific immunoglobulin (Ig)E, and lung histopathology were evaluated.

**Result:**

Tectorigenin significantly (P 〈 0.05) reduced eosinophil infiltration (41 ± 7%) in the broncho‐alveolar lavage fluid (BALF), serum IL‐5 level (41 ± 5, pg/mL), and bronchial and vascular inflammation (scores of 1.3 ± 0.2 and 1.1 ± 0.3, respectively) but had no significant effects on AHR, serum levels of IL‐33, −25, −13, and −4 (403 ± 24, 56 ± 7, 154 ± 11, and 89 ± 6 pg/mL, respectively), total and OVA‐specific IgE (2684 ± 265 and 264 ± 19 ng/mL, respectively), goblet cell hyperplasia, and mucus production.

**Conclusion:**

Tectorigenin could control inflammation and the secretion of inflammatory mediators of asthma, so it can be regarded as a potential antiasthma treatment with the ability to control eosinophilia‐related problems.

## Graphical abstract

Tectorigenin can control inflammation and inflammatory bio‐factors of asthma pathogens and can have a potential activity as anti‐inflammatory treatment in asthma and controlling of eosinophilia related problems.

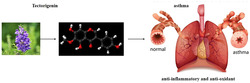

## 1 INTRODUCTION

Asthma is a respiratory disease with complicated pathophysiology and rising prevalence in the world, especially in developed countries. Asthma is characterized by airway hyperresponsiveness (AHR), airway inflammation, bronchoconstriction, and recurrent breathlessness. Allergy is the main factor triggering asthma attacks. In today's world, allergic asthma inflicts a high burden on public health and is a major contributor to morbidity and mortality caused by respiratory problems. Available antiasthma medications are not completely curative, and most of them merely control the complications of asthma.^1^, ^2^, ^3^

Asthma is orchestrated by allergic immune responses and an imbalance between T lymphocyte helper (Th) 1 and 2 responses, leading to dysregulated immune reactions in the lung, imbalanced production of proinflammatory and anti‐inflammatory factors, and respiratory complications.^4^, ^5^, ^6^ Inflammation contributes to physiological and also pathological immune processes, local vascular remodeling, and the recruitment of various immune cells. Epidemiological studies show that inflammation (chronic inflammation in particular) is linked with various diseases, including pulmonary diseases and asthma. Inflammation triggers the secretion of a series of proinflammatory cytokines, enzymes, and signaling proteins in affected tissues, such as asthmatic lungs. On the other hand, elevated levels of proinflammatory mediators are believed to expose patients to serious health threats in all stages of asthma. Thus, harnessing of inflammatory responses and signaling mediators is usually recognized as a potential therapeutic modality for asthma.^2^, ^5^, ^6^

Traditional Chinese medicine (TCM) has been used since ancient times to treat ailments and is recognized as an important source for pharmaceutical remedies. *Belamcanda chinensis* (L.) Redouté (Iridaceae) is used as a common herb in TCM to control inflammation, cough, pharyngitis, and tonsillitis. Recently, two isoflavones (tectorigenin and tectoridin) were isolated from this herb, both of which showed remarkable anti‐inflammatory activity. The role of tectorigenin in treating acute lung injury by suppressing inflammation has been investigated.^7^, ^8^, ^9^, ^10^ Tectorigenin is a methoxy‐isoflavone, in which the isoflavone is substituted by a methoxy group at position 6 and by hydroxy groups at positions 5, 7, and 4′. This plant metabolite, which is a member of 7‐hydroxy isoflavones, has been reported to be a suitable anti‐inflammatory agent.^7^, ^8^, ^9^, ^10^

In this study, we investigated the anti‐inflammatory, immunomodulatory, and antiasthma effects of tectorigenin in BALB/C mice models. Also, pathological changes in the respiratory system and airway allergic‐inflammatory responses were studied.

## 2 MATERIALS AND METHODS

### 2.1 Experimental animal model

BALB/c mice (n = 30 mice, male, 6–8 weeks old) were allocated to three groups: negative (healthy) control group (N), including nonasthmatic and nontreated mice, positive (asthmatic) control group (A), allergic asthma mice models, and tectorigenin‐treated group, which included asthmatic mice receiving tectorigenin via inhalation (T). During the study, all mice received food and water at libitum. In order to create asthma models, on days 1 and 14, the mice were sensitized by the intraperitoneal injection of OVA emulsified in aluminum hydroxide as the adjuvant. On days 24, 26, 28, and 30, the mice were challenged by the intratracheal delivery of OVA solution dissolved in phosphate‐buffered saline (PBS) for 30 min using a nebulizer.^5^, ^6^, ^11^ Mice in the negative control group only received normal saline following the same protocol. Treatments were delivered on days 25 and 27, and sampling was performed on day 31.

### 2.2 Assessment of AHR

In all groups, AHR was assessed to determine the enhanced pause (i.e., the Penh value). The AHR in methacholine (MCH) challenge test was conducted according to a previously described method.^5^, ^6^, ^11^ Briefly, for assessing AHR in mice in response to the MCH challenge test via intubation on day 31 and after tracheostomation, the trachea was connected to an inhalator, and increasing doses of MCH were inhaled to determine the Penh value.

### 2.3 Eosinophil enumeration

On day 31 (one day after the last challenge), the mice were euthanized and then tracheotomized, followed by BALF collection. First, BALF samples were cryo‐centrifuged; the supernatants were separated, and the cell sediment was used for cytospin slide preparation. The slides were stained, and differential cell counting was carried out to determine eosinophil percentage.^11^, ^12^, ^13^

### 2.4 Cytokines

The BALF supernatant was used to measure the levels of cytokines (IL‐33, IL‐25, IL‐13, IL‐5, and IL‐4) by the Bioplex‐Multiplex method.

### 2.5 Immunoglobulins

On day 31, blood samples were obtained from mice, and then sera were separated. The levels of total and OVA‐specific IgE were measured by specific ELISA kits (enzyme‐linked immunosorbent assay) following the provider's protocols.

### 2.6 Pathology

On day 31, lung tissues were obtained from mice, processed, and fixed on slides for preparing histopathological sections, which were stained with hematoxylin and eosin (H&E), Alcian blue‐periodic acid Schiff (AB‐PAS), PAS, and AB‐PAS‐H&E stains. The slide sections were evaluated by light microscopy to examine for infiltration of eosinophils around bronchi and vessels, meta‐hyperplasia of goblet cells, and mucus hypersecretion.^5^, ^6^, ^11^, ^12^, ^13^

### 2.7 Statistical analysis

Data were expressed as mean ± standard deviation (SD). SPSS software was used for statistical analyses. Data were analyzed by the paired *t* test to determine if differences between groups were statistically significant. A *P* value of <0.05 was supposed to be statistically significant. For drawing graphs, GraphPad Prism software was used.

## 3 RESULTS

### 3.1 AHR

**FIGURE 1:**
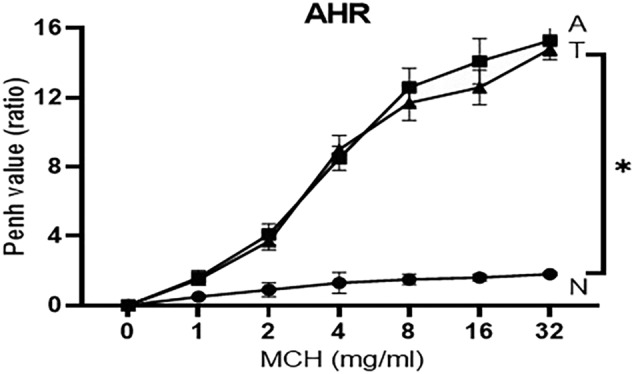
Airway hyperresponsiveness (AHR). The Penh value in the MCH challenge test was determined in all three groups, and AHR was assessed in response to MHC. The symbol * shows that there was a significant difference between tectorigenin‐treated and nontreated asthma groups, indicating a significant decrease in AHR in tectorigenin‐treated mice (*P* < 0.05).

The Penh value (an indicator of AHR) was significantly (*P* < 0.05) increased in asthmatic control mice compared with healthy animals (Figure 1) at all MCH concentrations. However, the Penh value nonsignificantly decreased in asthmatic mice treated with tectorigenin compared with asthmatic nontreated animals (*P* > 0.05).

### 3.2 Eosinophil percentage in BALF

**FIGURE 2:**
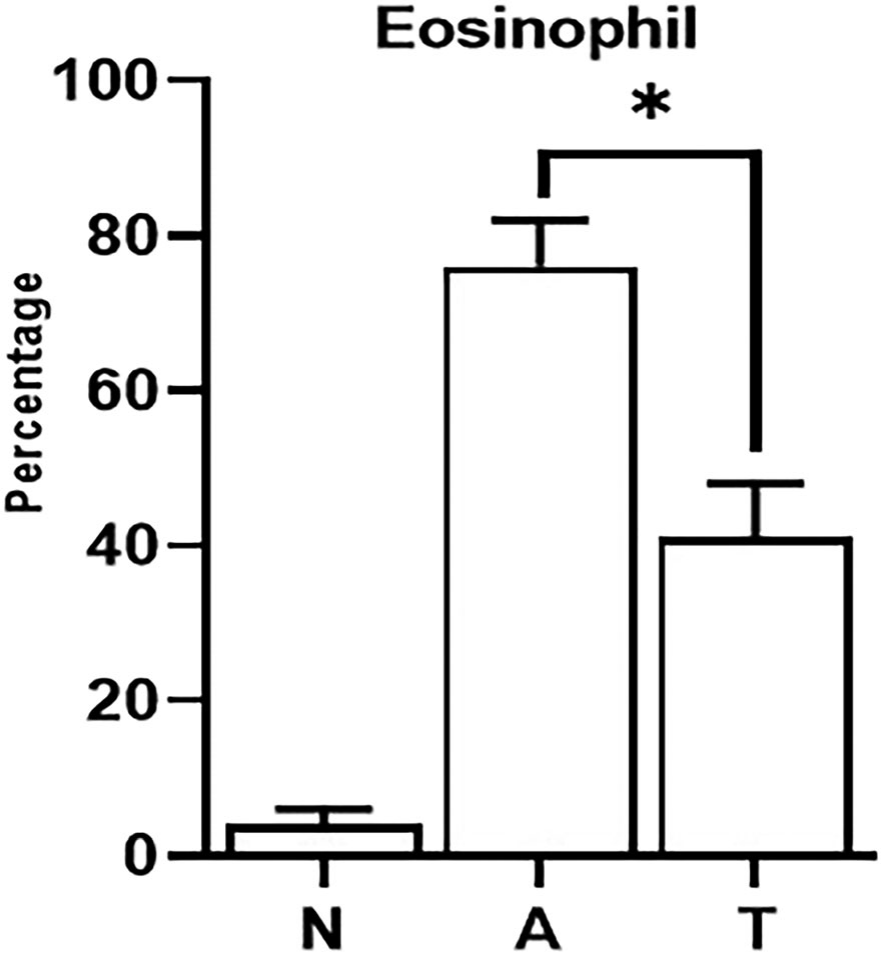
Eosinophils in BALF samples. The percentage of eosinophils in BALF was determined in all study groups via conventional staining and differential counting by microscopy. The symbol * shows that there was a significant difference between tectorigenin‐treated (T) and nontreated asthma (A) groups in such a way that the percentage of eosinophils in BALF was significantly lower in tectorigenin‐treated mice (*P* < 0.05) compared with asthma group.

The percentage of eosinophils in BALF was significantly higher in asthmatic mice (76 ± 6%) compared with control healthy mice (4 ± 2%) (*P* < 0.05). Treatment with tectorigenin significantly decreased eosinophil percentage in asthmatic mice (41 ± 7%, *P* < 0.05) (Figure 2).

### 3.3 Cytokines

**FIGURE 3:**
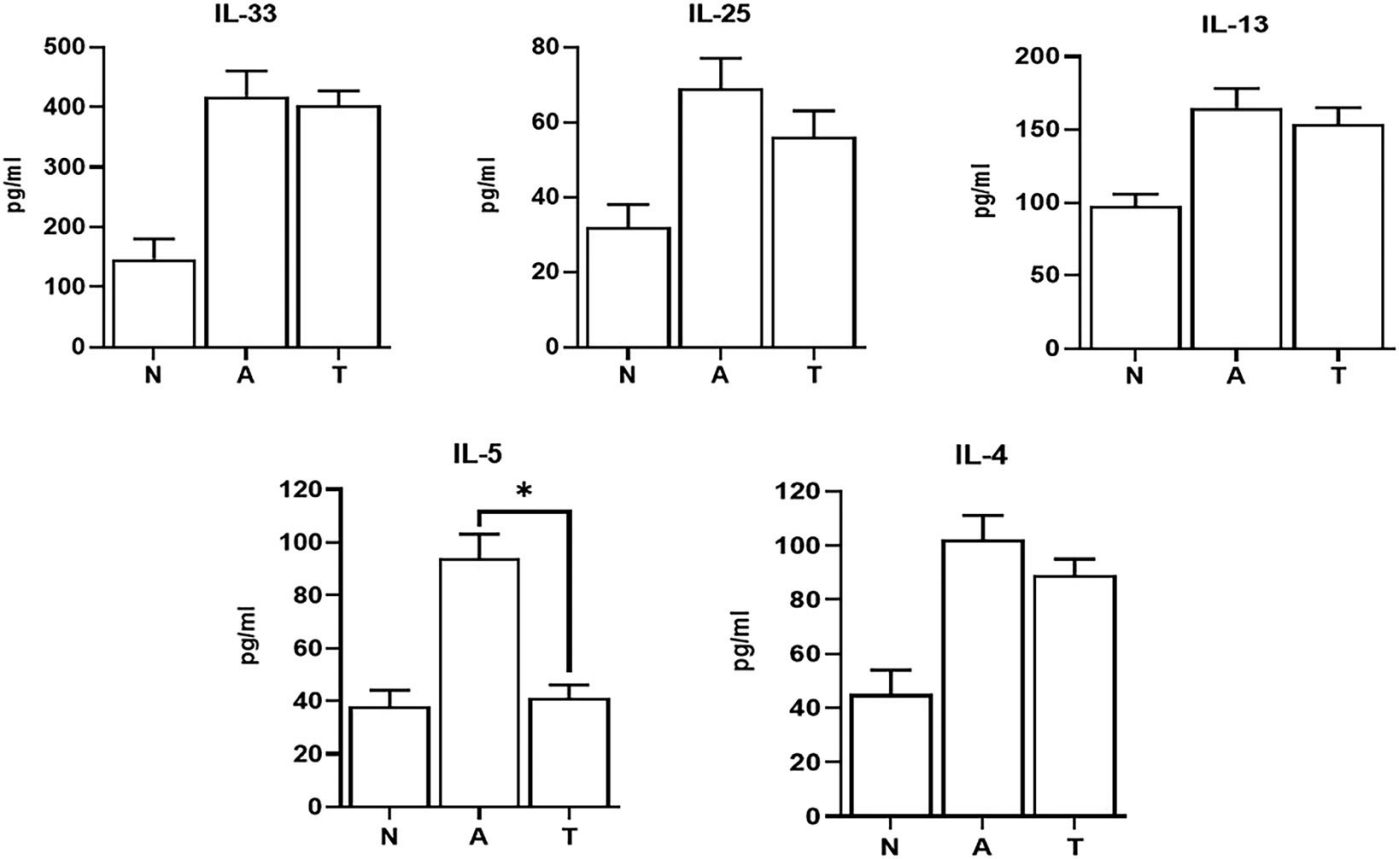
Cytokines levels in BALF. The levels of the main cytokines involved in allergic responses, IL‐33, IL‐25, IL‐13, IL‐5, and IL‐4, were measured in BAL fluid samples. The symbol * shows that there was a significant difference comparing IL‐5 levels between tectorigenin‐treated (T) and nontreated asthma (A) groups, indicating that BALF IL‐5 level was significantly lower in tectorigenin‐treated mice (*P* < 0.05) compared with asthma group. Other cytokines were decreased in tectorigenin‐treated group compared with asthma group, but the decreasing was not significant.

The levels of IL‐33, IL‐25, IL‐13, IL‐5, and IL‐4 cytokines in BALF significantly increased in asthmatic animals compared with healthy controls (*P* < 0.05). In asthmatic mice treated with tectorigenin, the levels of these cytokines were significantly reduced (403 ± 24, 56 ± 7, 154 ± 11, 41 ± 5, and 89 ± 6 pg/mL, respectively); however, declines in IL‐33, IL‐25, IL‐13, and IL‐4 were not significant compared with nontreated asthmatic mice (Figure 3). On the other hand, the decrease in IL‐5 following tectorigenin treatment was statistically significant compared with control asthmatic animals (*P* < 0.05).

### 3.4 IgE

**FIGURE 4:**
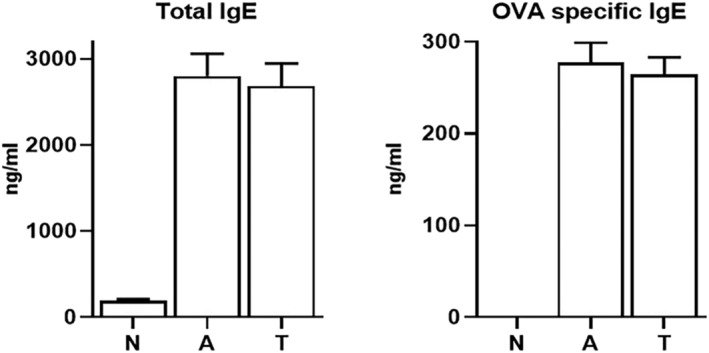
Serum total and OVA‐specific immunoglobulin E levels. The levels of total and OVA‐specific IgE were measured in the sera samples of all mice. The two IgEs were decreased in tectorigenin‐treated group compared with asthma group, but the decreasing was not significant.

The serum levels of total and OVA‐specific IgE were significantly (*P* < 0.05) increased in asthmatic control mice compared with healthy animals (Figure 4). Treatment of asthmatic mice with tectorigenin attenuated the increase in the serum levels of total and OVA‐specific IgE (2684 ± 265 and 264 ± 19 ng/mL, respectively), but not to a statistically significant level compared with nontreated asthmatic control animals (*P* > 0.05).

### 3.5 Histopathology

**FIGURE 5:**
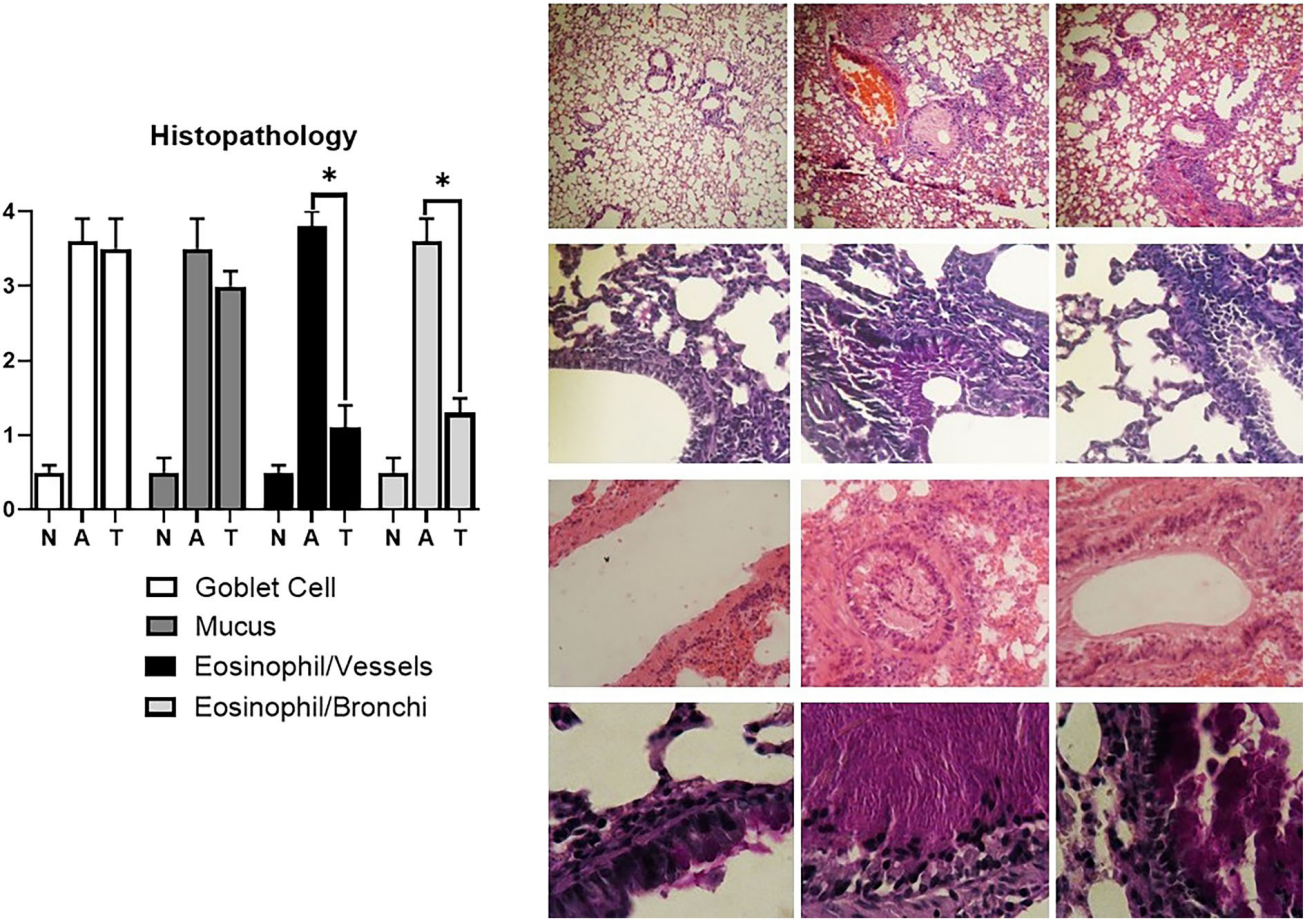
Lung histopathology examination. Inflammation with eosinophil infiltration around bronchi and vessels, metaplasia of goblet cells, and hyper‐production of mucus were studied in lung tissue sections using H&E, AB‐PAS, PAS, and AB‐PAS‐H&E staining. The symbol * shows that there was a significant difference between tectorigenin‐treated and nontreated asthma groups. Eosinophilic infiltration around bronchi and vessels was significantly lower in the lung tissues of tectorigenin‐treated mice (*P* < 0.05). In pathologic figures, the first column belongs to N, the second column belongs to A, and the third column belongs to T.

In histopathologic examination of the lung tissue, infiltration of eosinophils around bronchi and vessels, goblet cell hyperplasia, and mucus production were prominent in the asthmatic group compared with the healthy control group (*P* < 0.05 for all; Figure 5). However, tectorigenin treatment considerably attenuated eosinophil infiltration around bronchi and vessels (scores of 1.3 ± 0.2 and 1.1 ± 0.3, respectively) in asthmatic mice compared with the positive control group. Also, the goblet cell hyperplasia index and mucus production were reduced in the tectorigenin‐treated group compared with the positive control group, but the differences were not statistically significant (*P* > 0.05).

## 4 DISCUSSION

Asthma represents a pathophysiologically heterogeneous disease characterized by airway inflammation. Aeroallergens are the main triggers of IgE‐mediated allergic asthma. Most patients with allergic asthma suffer from concomitant comorbidities, for example, atopic dermatitis, allergic rhinitis, and food allergies.^14^, ^15^ Inhaled allergens induce type‐2 inflammatory responses, leading to the symptoms of allergic asthma and, subsequently, tissue remodeling due to persistent allergen exposure.^16^ Allergen immunotherapy for allergic rhinitis has greatly reduced the prescription of medications for relieving allergic asthma, as well as hospitalization due to symptom exacerbation.^17^, ^18^ Allergic asthma is the most common phenotype of asthma, which is defined by sensitization due to exposure to environmental allergens. Atopic dermatitis and allergic rhinoconjunctivitis are particularly common in patients with allergic asthma. Allergic asthma is clinically milder than nonallergic asthma, which is associated with higher levels of total IgE and Th2 cytokines in allergic versus nonallergic asthma.^19^ Conventional treatments available for allergic pulmonary diseases restore immune tolerance to allergens and attenuate early‐ and late‐onset allergen‐specific airway hyper‐reactivity, causing disease remission and preventing from new‐onset sensitizations.^20^ In this study, we observed that tectorigenin treatment reduced the levels of main allergic and inflammatory cytokines (IL‐33, IL‐25, IL‐13, IL‐5, and also IL‐4) in BALF in asthmatic mice; however, only the decrease in IL‐5 level was statistically significant. Therefore, tectorigenin seems to be able to control allergic inflammation through suppressing IL‐5 production and alleviating eosinophilic inflammation.

Isoflavonoids are members of a broad class of plant‐derived polyphenols. These compounds manifest wide‐range activities such as antioxidant and anti‐inflammatory properties.^21^ Tectorigenin, a flavonoid, has been shown to have immunomodulatory activities partly by inhibiting nitric oxide (NO) production and inducible‐NO synthase (iNOS) expression.^22^ The release of NO is induced during inflammatory responses, and inflammatory cytokines such as interferon (IFN)‐γ have been noted to regulate the activity of iNOS in macrophages.^23^ Tectorigenin was reported to decrease the level of IL‐1β mRNA, one of the most potent proinflammatory cytokines. Moreover, IL‐1 is an important contributor to inflammatory diseases such as asthma by persistently inducing the gene expression of cyclooxygenase (COX)‐2 and prostaglandin (PG)E2 synthase. In addition, IL‐1β stimulates NO release and iNOS expression.^8^, ^24^ The expression of NO, iNOS, COX‐2, and proinflammatory cytokines requires the activation of the nuclear factor kappa B (NF‐κB), a mammalian transcription factor regulating the expression of a variety of genes involved in cytokine‐induced immunity and inflammation.

Tectorigenin inhibits IFN‐γ/lipopolysaccharide (LPS)‐induced NF‐κB activation by facilitating the degradation of the inhibitor of kappa B (IκB)‐α, suggesting an anti‐inflammatory role for this compound.^8^, ^25^ Tectorigenin has also been noted to inhibit the production of PGE2 in peritoneal macrophages, which is consistent with its anti‐inflammatory role by suppressing COX‐2 induction and PGE2 production in stimulated inflammatory cells.^7^, ^26^ In the current study, we noticed that AHR (represented by the Penh value) was decreased in asthmatic mice treated with tectorigenin; however, this effect was not statistically significant. Therefore, tectorigenin seems to have partial effects on AHR.

In a study, tectorigenin was noted to decrease the recruitment of neutrophils to the lung tissue after LPS exposure, evidenced by decreased myeloperoxidase (MPO) activity.^9^ Eosinophilic inflammation is a dominant feature of allergic asthma, and controlling eosinophilic infiltration is a main key in asthma management. In this study, tectorigenin was observed to markedly reduce eosinophil infiltration around pulmonary bronchi and vessels in asthmatic mice, suggesting that tectorigenin can be a potential inhibitor of eosinophilic inflammation in asthma.

As one of the main proinflammatory cytokines, IL‐6 is produced by various inflammatory cells such as T and B lymphocytes, macrophages, fibroblasts, and endothelial and epithelial cells. It can also be released by airway muscle cells under IL‐1β stimulation. Elevation of IL‐6 levels is an important biomarker of inflammation. Also, IL‐8, a specific chemokine and activator of neutrophils, plays an important role in regulating inflammation. Treatment with tectorigenin could downregulate these inflammatory mediators in OVA‐induced asthma animal models in a dose‐dependent manner. Pulmonary fibrosis is a feature of OVA‐sensitized asthma animal models, which might lead to pulmonary dysfunction. The loss of lung function due to fibrosis is common in asthma patients, and high‐dose tectorigenin could inhibit pulmonary fibrosis, which can be partly attributed to inducing the expression of vascular endothelial growth factor A (VEGFA), tumor necrosis factor (TNF)‐α, and IFN‐c.^10^, ^27^, ^28^ Another regulatory mechanism through which tectorigenin might inhibit pulmonary fibrosis can be orchestrated by modulating the transforming growth factor (TGF)‐b1/Smad and toll‐like receptor (TLR)‐4/NF‐ĸB signaling pathways.^10^ We observed that inflammation caused by eosinophils' accumulation around bronchi and vascular beds was significantly attenuated by tectorigenin, but tectorigenin could not significantly reduce goblet cell hyperplasia and mucus production. These findings showed that tectorigenin could act as an anti‐inflammatory but not anti‐mucolytic agent.

Tectorigenin shows strong antioxidant effects, evidenced by assays such as xanthine–xanthine oxidase, 2,2‐diphenyl‐1‐picrylhydrazyl (DPPH), 1,1‐diphenyl‐2‐pirylhydrazyl radical scavenging, superoxide anion radical removal, and lipid peroxidation.^29^, ^30^, ^31^ As an anti‐inflammatory agent, tectorigenin inhibits the expressions of iNOS and COX‐2 and the production of PGE2 and NO, as well as IL‐1β secretion.^7^, ^8^ Tectorigenin offers a promising candidate for drug development because it has shown no acute or subacute toxicity.^29^, ^32^ Tectorigenin has also exhibited analgesic effects mediated by the inhibition of the synthesis of arachidonic acid metabolites through blocking COX‐2.^7^, ^8^ Carrageenan‐induced paw edema is a model for studying inflammation as it involves the release of different mediators in distinct phases, including histamine and serotonin in the first phase, and kinin and prostaglandins in the subsequent phase, all of which were inhibited by tectorigenin.^33^, ^34^ In the recent years, effect of similar biomolecules on asthma was investigated and notable results were observed.^35^, ^36^ In this study, serum IgE levels (total and OVA‐specific) were reduced in asthmatic mice treated with tectorigenin; however, this decrease was not statistically significant. This observation suggested that tectorigenin might not act as a potent anti‐allergic agent and promoted no significant effects on asthma pathogenesis via the allergic pathways mediated by IgE. However, our study showed that tectorigenin could act as a potent anti‐inflammatory factor by suppressing proinflammatory cytokines in mice models of allergic asthma. Therefore, tectorigenin can be regarded as an anti‐inflammatory agent to control airway inflammation in asthma, but this notion needs more investigations to be confirmed.

## AUTHOR CONTRIBUTIONS

Jingning Guo, Yanping Shi, Yujun Wang, and Tao Chen participated in the planning, study, testing, analysis of data, and writing. Seyyed Shamsadin Athari participated in analysis of data, editing and revising of the manuscript.

## CONFLICT OF INTEREST STATEMENT

There is no conflict of interest.

## ETHICS STATEMENT

This research was approved by the ethic committee of animal house of ix.med.vet.dep, 2023 (No. IX.MED.VET.DEP.REC.2023.0200003.6).

## Data Availability

Data are available upon request from the corresponding author.
